# Pyrrolocin C and equisetin inhibit bacterial acetyl-CoA carboxylase

**DOI:** 10.1371/journal.pone.0233485

**Published:** 2020-05-29

**Authors:** Erica C. Larson, Albebson L. Lim, Christopher D. Pond, Matthew Craft, Mirela Čavužić, Grover L. Waldrop, Eric W. Schmidt, Louis R. Barrows

**Affiliations:** 1 Department of Pharmacology and Toxicology, University of Utah, Salt Lake City, Utah, United States of America; 2 Department of Biological Sciences, Louisiana State University, Baton Rouge, Louisiana, United States of America; 3 Department of Medicinal Chemistry, University of Utah, Salt Lake City, Utah, United States of America; University of British Columbia, CANADA

## Abstract

Antimicrobial resistance is a growing global health and economic concern. Current antimicrobial agents are becoming less effective against common bacterial infections. We previously identified pyrrolocins A and C, which showed activity against a variety of Gram-positive bacteria. Structurally similar compounds, known as pyrrolidinediones (e.g., TA-289, equisetin), also display antibacterial activity. However, the mechanism of action of these compounds against bacteria was undetermined. Here, we show that pyrrolocin C and equisetin inhibit bacterial acetyl-CoA carboxylase (ACC), the first step in fatty acid synthesis. We used transcriptomic data, metabolomic analysis, fatty acid rescue and acetate incorporation experiments to show that a major mechanism of action of the pyrrolidinediones is inhibition of fatty acid biosynthesis, identifying ACC as the probable molecular target. This hypothesis was further supported using purified proteins, demonstrating that biotin carboxylase is the inhibited component of ACC. There are few known antibiotics that target this pathway and, therefore, we believe that these compounds may provide the basis for alternatives to current antimicrobial therapy.

## Introduction

Antimicrobial resistance is a growing public health concern worldwide [1, 2]. Compounds derived from natural sources offer novel chemistry and may provide a solution to drug resistance. Most approved drugs currently available are derived from natural products or inspired by natural product pharmacophores. From the years 1981–2019, nearly 50% of small molecules approved by the FDA were natural products [3].

Fungal natural products from the class known as pyrrolidinediones are often antibacterial in the low micromolar range, yet their mechanisms of action are unknown. We previously identified pyrrolocin A (PYRA), which was isolated from a phylogenetically novel strain of endophytic fungus from Papua New Guinea (NRRL accession number 50135) and possessed moderate activity against the attenuated strain of *Mycobacterium tuberculosis* (Mtb), H37Ra [4]. A heterologous expresser was developed to improve production, yielding desmethyl isomers of pyrrolocin A: pyrrolocins B and C (PYRB and PYRC) [5]. In addition, pyrrolocins A-C were selectively active against additional Gram-positive bacteria including *S*. *aureus*, *Streptococcus pneumonia*, and *Bacillus subtilis*. Other pyrrolidinediones, including TA-289 and equisetin (EQI), also display antibacterial activity against a wide range of pathogens in a single-digit μg/mL range [6, 7]. However, the mechanism of action of these compounds against bacteria was undetermined.

**Fig 1:**
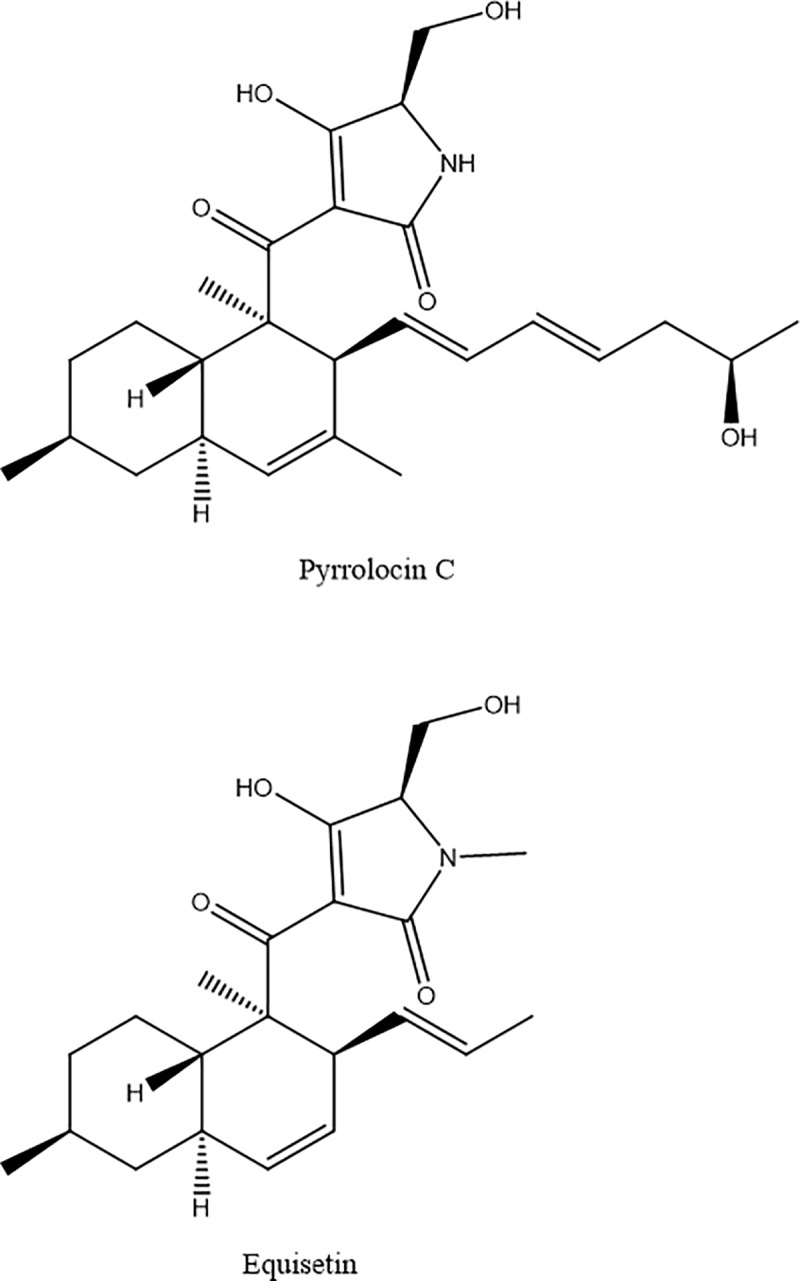
Chemical structures of PYRC (top) and EQI (bottom).

Here, we investigate the mechanism by which PYRC and EQI (Fig 1) exert toxicity in *S*. *aureus*, MRSA and additional drug resistant strains. We describe the initial transcriptomic and metabolomic approaches used to inform our hypothesis that PYRC and EQI inhibit fatty acid biosynthesis by targeting the bacterial acetyl-CoA carboxylase. Fatty acid rescue, acetate tracing and enzyme inhibition studies indicate that biotin carboxylase is catalytic domain inhibited by PYRC and EQI. There are few known antibiotics that target this pathway and, therefore, we believe that these compounds may provide interesting alternatives to current antimicrobial therapy.

## Results

### PYRC and EQI broadly inhibit bacterial growth

PYRC and EQI showed activity against a broad panel of Gram-positive bacteria and mycobacteria, but were inactive against Gram-negative bacteria. Both compounds showed IC_90_s of 1 μg/mL against *S*. *aureus*, including methicillin-sensitive and methicillin-resistant strains (Table 1 and S1 Fig). PYRC and EQI were active against an expanded panel of doxycycline, clindamycin or vancomycin resistant *S*. *aureus* and *E*. *faecalis* strains (Table 1 and S1 Fig), and in previous work similar activity was found for PYRC against *M*. *tuberculosis*.

**Table 1:** IC_90_ (μg/mL) and IC_50_ data for pyrrolocin B, PYRC and EQI.

		IC_90_ (μg/mL)				IC_50_ (μg/mL)
	*S*. *aureus* (ATCC 12600)	Methicillin-resistant *S*. *aureus* (ATCC 43300)	Clindamycin and Doxycycline-resistant *S*. *aureus* (CDC-568)	Vancomycin-resistant *E*. *faecalis* (ATCC 51229)	*E*. *coli* (ATCC 23724)	CEM-TART (1A2)
pyrrolocin B	16	16	16	>32	*nt*	<32
pyrrolocin C	1	1	0.5	2	>128	8.841
equisetin	1	1	1	2	>128	9.692
polymyxin B	*nt*	*nt*	*nt*	*nt*	0.5	*nt*
polymyxin B nonapeptide	*nt*	*nt*	*nt*	*nt*	>64	*nt*

IC_90_’s determined as concentration required for > 90% inhibition, each determination performed at least twice, in quadruplicate. CEMTART (1A2) is a CCRF-CEM T lymphoblastic leukemia cell line derivative [11]. *nt* is not tested. IC_50_ experiment repeated twice, each concentration performed in triplicate.

**Fig 2:**
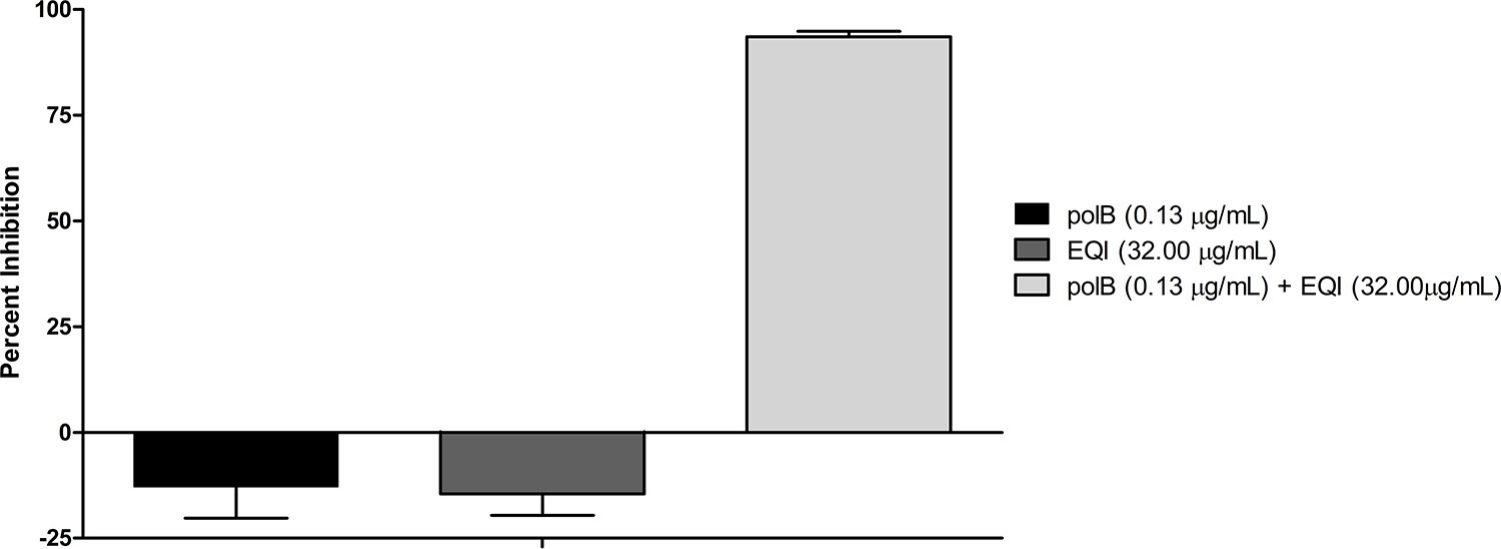
Potentiation of EQI treated in combination with polymyxin B. Percent inhibition of polymyxin B (0.125 μg/mL) and equisetin (32 μg/mL) treated singly and in combination against *E*. *coli* (ATCC 23724). Polymyxin B is represented by “polB”.

Because of this activity pattern, we suspected that drug penetration might be responsible for the lack of activity against Gram-negative bacteria. To test this, we measured the synergistic effect of EQI and PYRC with the outer membrane-permeabilizing agents polymyxin B and polymyxin B nonapapetide (Fig 2 and S2 Fig) [8]. When the membrane was permeabilized, EQI and PYRC both killed *Escherichia coli*. There was a significant potentiation effect (>90% inhibition) when EQI (32 μg/mL) was tested together with polymyxin B (0.125μg/mL) (Fig 2). Polymyxin B did not potentiate PYRC, but limited potentiation was observed using the nonapeptide. A maximum inhibitor effect of ~30% was achieved when PYRC (128 μg/mL) was combined with polymyxin B nonapeptide (8 μg/mL) (S2 Fig). As we show below, this result is consistent with the relative potency of EQI and PYRC against the molecular target in *E*. *coli*.

Toxicity testing against a drug sensitive human cell line (CEMTART 1A2 [9]) yielded comparable therapeutic indices (TI) for the two compounds (Table 1 and S1 Fig).

### PYRC induces pathways associated with energy metabolism in Mtb

**Fig 3:**
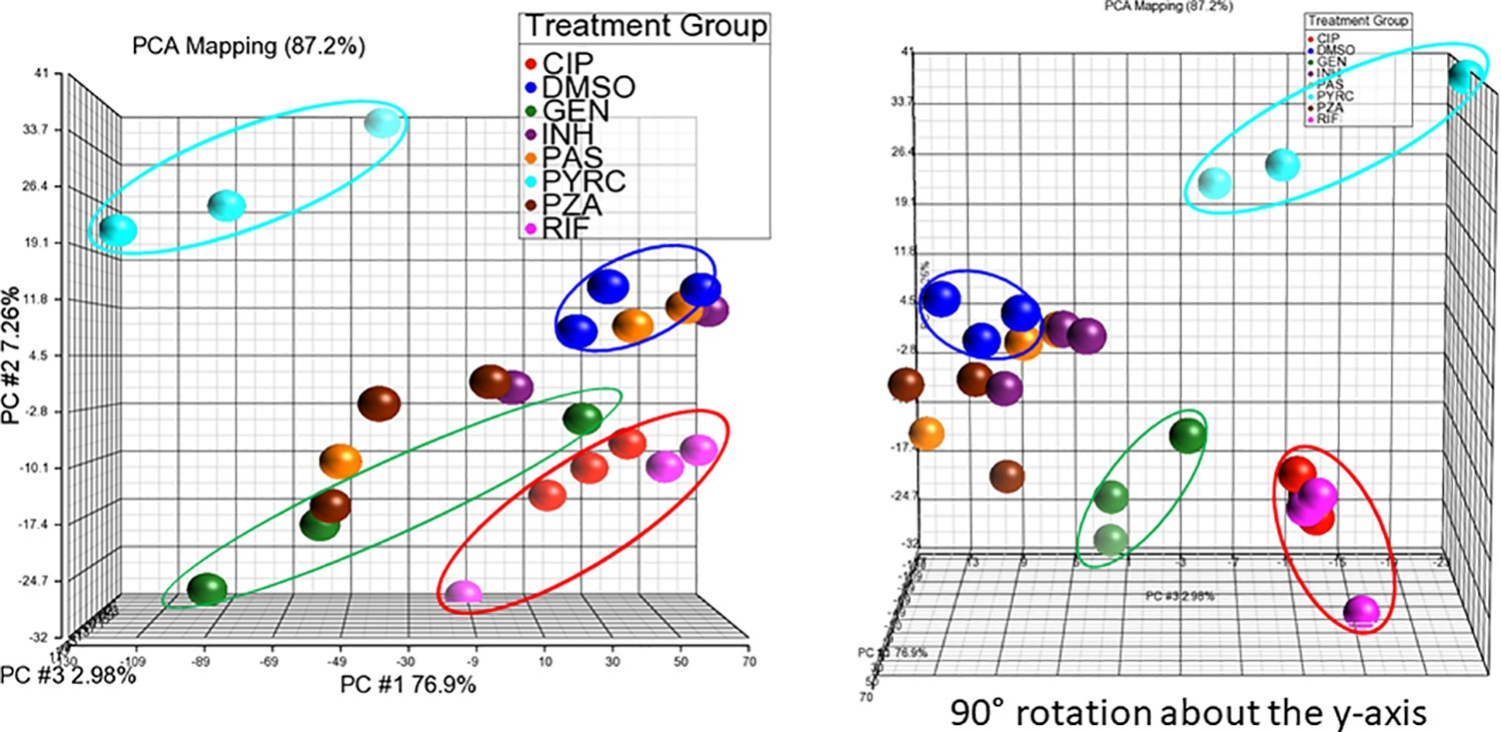
PCA of transcriptomic responses of Mtb cultures to anti-TB drug treatments. Mtb cultures were treated in triplicate with ciproflaxin (red), DMSO (blue), gentamicin (green), isoniazid (purple), para-aminosalicylic acid (yellow), PYRC (cyan), pyrazindamide (brown), or rifampicin (magenta) for 6 hours at 10X IC_90_ or IC_90_. Vehicle (DMSO) cluster (circled in blue); transcription synthesis inhibitor cluster (circled in red); protein synthesis inhibitor cluster (circled in green); and PYRC cluster (circled in cyan). RNA-seq was performed and data analysis was completed in Partek®Genomic Suite®.

PYRC was used for preliminary transcriptomic analysis in *M*. *tuberculosis* (H37Ra), since transcriptomic changes due to drug treatment have previously been used to reveal a drug’s mechanism of action against Mtb [10–12]. Based upon this previous work (Dr. Helena Boshoff, personal communication), we used high concentrations of drug (IC_90_ or 10X IC_90_), tested in triplicate, over a 6-hour exposure time course. We used a panel of known anti-tuberculosis drugs and compared them with PYRC. Using principal component analysis (PCA) of the transcriptomes, we found that individual drugs created visibly different clusters (Fig 3). This reaveled that each drug created a different set of transcriptional changes, indicating that each had different mechanisms of drug action. As expected, the transcription component inhibitors rifampin and ciprofloxacin, formed a cluster when compared to the other drug treatments (circled in red; Fig 3). Gentamicin, a protein synthesis inhibitor, also formed a cluster spatially separate from all other treatments (circled in green; Fig 3). Isoniazid, para-aminosalicylic acid, and pyrazinamide did not form distinct clusters comparative to vehicle (DMSO, circled in blue; Fig 3) indicating that under our experimental conditions, these drugs did not elicit a consistent effect on Mtb. These drugs may require a longer incubation period in order to induce consistent effects on the transcriptome. The most striking feature that resulted from this analysis was that PYRC occupied an entirely separate region of the PCA plot from the known drugs, suggesting a unique transcriptomic signature (circled in cyan; Fig 3).

Pathway analysis of transcriptomic changes using the Database for Annotation, Visualization and Integrated Discovery (DAVID) [13] revealed that PYRC most impacted oxidative phosphorylation (S1 Table). We speculate that this might be mechanistically related to the reported activity of an EQI analog, TA-289, which causes aberrant mitochondrial morphology in yeast that is not due to reactive oxygen species, inhibition of cardiolipin synthesis, or direct effects on the electron transport chain [6].

Other transcription signatures that were significantly affected by PYRC treatment include (in order of decreasing enrichment score): ATP binding, pyrimidine metabolism, cation binding, and ribosomal protein processes (S1 Table). The meaning of these changes was difficult to discern. Ultimately, we attributed them to metabolic shifts and stress responses caused by PYRC. For example, transcripts of pathways in pyrimidine metabolism were upregulated. Protein synthesis inhibitors upregulate enzymes involved in pyrimidine metabolism, possibly to conserve nucleotides during translation inhibition [10, 11]. Similarly, if energy metabolism were impeded by PYRC, induction of salvage pathways may be a compensatory response to reduced function of vital pathways, such as translation. However, one transcription process noted as a possible indicator of PYRC mechanism of action was cation binding, more specifically metal ion binding. Metal binding has been suggested as an important feature of EQI bioactivity [14–16].

### EQI antibacterial activity not affected by metal ion concentration

**Fig 4:**
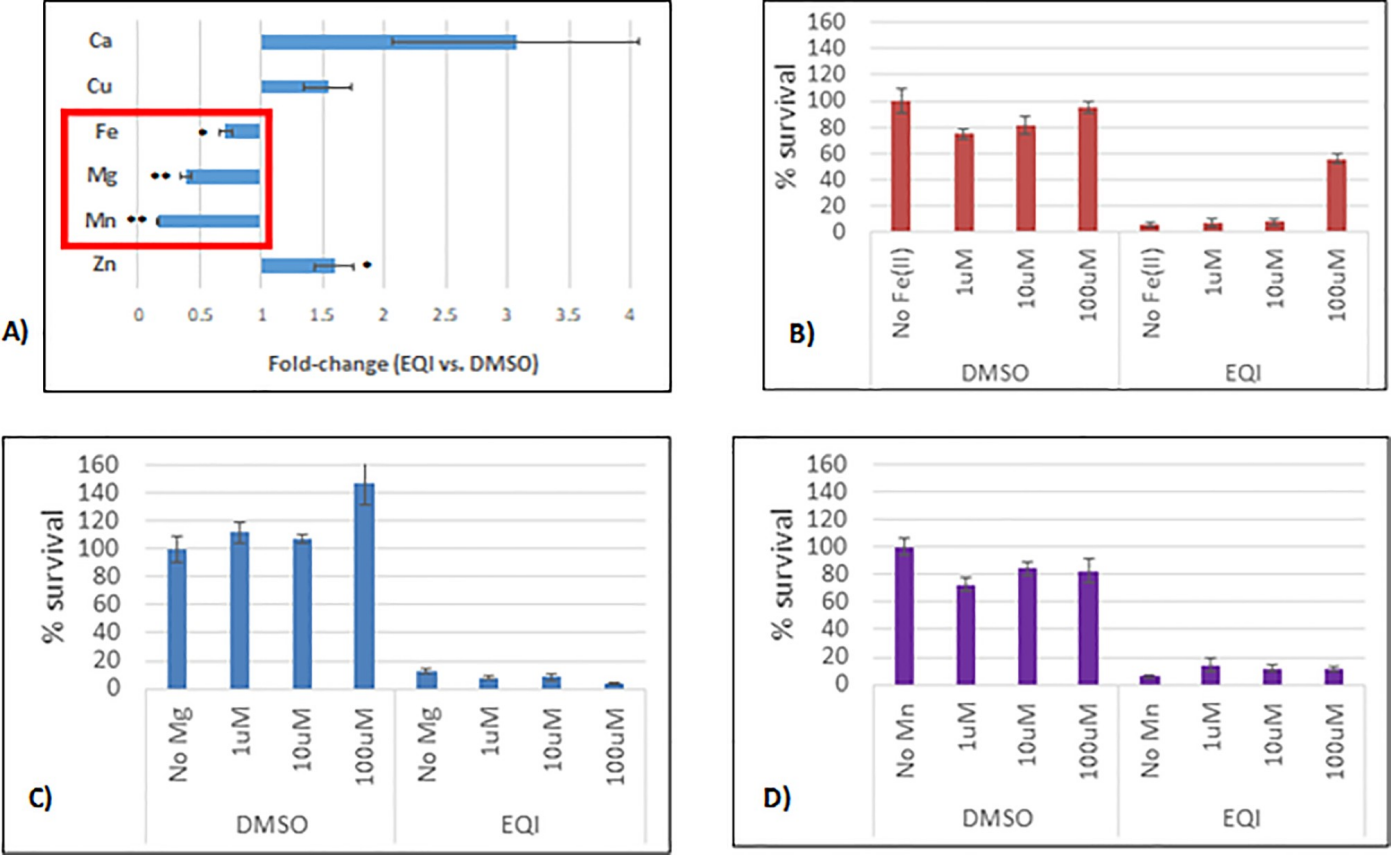
Total metal analysis of EQI-treated *S*. *aureus* by ICP-OES and metal rescue. Panel **A),** metal analysis of equisetin-treated *S*. *aureus S*. *aureus* culture was added to LB broth, with DMSO or EQI (IC90; 4.36 μM). Cultures were incubated at 37°C for 2 hours, digested in nitric acid (trace metal grade) and analyzed by ICP-OES. Total metal concentrations were adjusted based on final OD_600_. Co, Cd, Ni, Ag, & Se total metal concentrations < Limit of Detection. Mean ± SEM, n = 11. *p<0.05; **p<0.005 (Student’s t-test). Panel **B),** *S*. *aureus* cultures were incubated in DMSO or EQI (IC90; 4.36 μM) in the presence or absence of exogenous metals. Metal solutions (MgCl, MnCl, & Fe(EDTA)) were: 1 μM, 10 μM, and 100 μM. Percent survival determined by the MTT assay. Mean ± SEM, n = 6. *p<0.05 (Student’s t-test).

Based upon the published data and transcriptome indicators, we explored metal homeostasis in EQI treated *S*. *aureus*. Tetramic acids are known to bind metal ions [16]. Upon proton loss, the tetramate anion has several resonance forms that promote bidentate interactions with metals [15]. In *S*. *aureus* metal homeostasis assessed by inductively-couple plasma–optical emission spectroscopy (ICP-OES), we observed EQI treatment to induce significant decreases in endogenous levels of iron, magnesium, and manganese, while causing an increase in copper levels (Fig 4A). These findings might indicate that the drug sequesters metal or inhibits metal ion transport. We then attempted to rescue the bacteria from potential effects of such metal sequestration by supplementing the media with exogenous metal ions (Fig 4B). We found no protection was conveyed against EQI (IC_90_) at any of the test concentrations for magnesium or manganese. Furthermore, we found only moderate protection (~55%) at high, non-physiological, concentrations of iron. Metals did not increase PYRC toxicity. This result stood in contrast to previous work on an anti-TB drug with metal dependent-toxicity corresponding with transcription signatures of metal metabolism [10]. Since neither rescue nor toxic exacerbation could be achieved with exogenous metal ions, it seemed unlikely that metal sequestration or dysregulation of metal metabolism was responsible for *S*. *aureus* inhibition.

### EQI decreases malonyl-CoA levels in *S*. *aureus*

A transposon *S*. *aureus* library [17] was screened for resistance to EQI, but no informative hits were observed. Metabolomic analysis of EQI-treated *S*. *aureus* lysates was therefore used to gain insight into mechanism of action. Several changes in key intracellular metabolites were observed. Tricarboxylic acid cycle (TCA) intermediates as well as amino acids were elevated (Table 2), which indicates a stress response in *S*. *aureus* due to addition of antibiotics [10, 18, 19]. Consistent with an increase in TCA intermediates, which is an NAD-consuming and NADH-generating cycle, there was a downward trend in NAD levels and upward trend in NADH levels in response to EQI. Critically, malonyl-CoA was significantly decreased following EQI treatment. Malonyl-CoA is the product of acetyl-CoA carboxylase, the first committed step in fatty acid biosynthesis. Also, NADP levels were decreased and NADPH levels were increased, suggesting reduced fatty acid biosynthesis. Taking these findings together, we proposed the hypothesis that EQI and PYRC inhibit fatty acid biosynthesis in *S*. *aureus*.

**Table 2:** EQI decreases malonyl-CoA levels in *S*. *aureus*.

	Fold-change (EQI vs. DMSO)		p-value
**Malonyl-CoA**	▼	0.20	<0.01
**AMP**	▼	0.27	0.01
**NAD+**	▼	0.48	0.16
**ADP**	▼	0.71	0.01
**ATP**	▼	0.79	0.14
**GDP**	▼	0.87	0.50
**NADP+**	▼	0.91	0.62
**Adenine**	▼	0.94	0.70
**Pyruvate**	▼	0.96	0.94
**Acetyl-CoA**	▲	1.21	0.26
**Glycerol-3P**	▲	1.31	0.20
**NADH**	▲	1.37	0.70
**Glutamate**	▲	1.38	<0.01
**BSSB**	▲	1.42	0.21
**CoASH**	▲	1.49	0.38
**Proline**	▲	1.56	<0.01
**Alanine**	▲	1.60	<0.01
**Succinate**	▲	1.67	<0.01
**BSH**	▲	1.70	<0.01
**Glutamine**	▲	1.79	<0.01
**Fumarate**	▲	1.95	<0.01
**GTP**	▲	2.11	0.01
**NADPH**	▲	3.24	0.07
**Adenosine**	▲	4.21	<0.01
**Malate**	▲	5.52	0.03
**Lactate**	▲	7.13	0.07

*S*. *aureus* cultures were treated with EQI (4.36 μM) for 2 h in quintuplicate. Metabolites were quantified by LC-MS. Metabolites were normalized to bacterial concentrations and fold-changes were determined in comparison to DMSO. Significance of fold-change was determined using a two-tailed, unpaired t tests; DMSO vs EQI. Analytes with a p < 0.05 are indicated in red.

### Exogenous fatty acids prevent growth inhibition by PYRC and EQI in *S*. *aureus*

**Fig 5:**
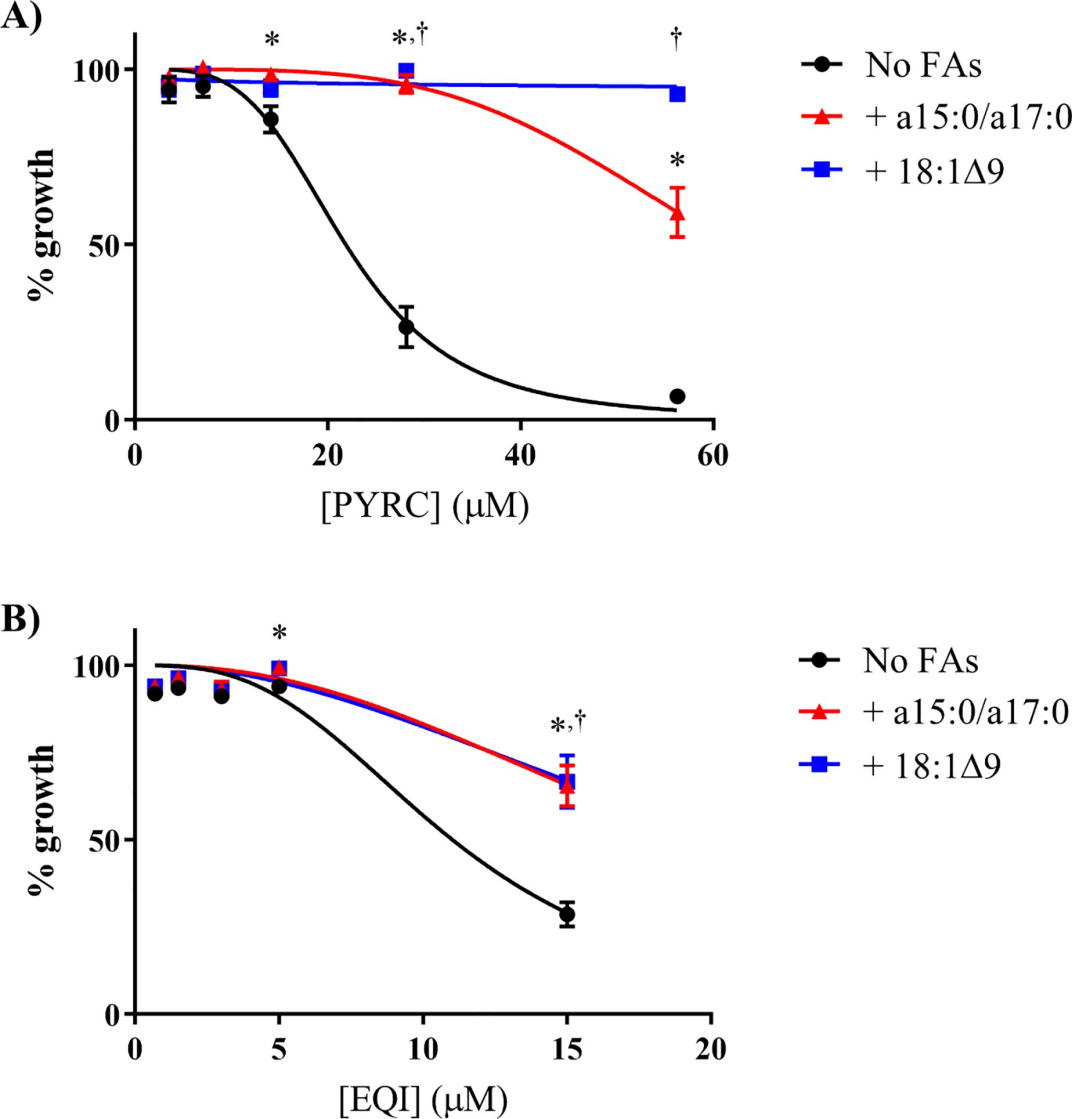
Fatty acid supplementation can overcome growth inhibition by PYRC and EQI treatment in *S*. *aureus*. *S*. *aureus* was grown in the presence of drug in LB+BSA (No FAs; black circle), LB+BSA plus a15:0/a17:0 (500 μM, 2:1 ratio of a15:0 to a17:0; red triangle), or LB+BSA plus 18:1Δ9 (500 μM; blue square). Mean data of two independent experiment run in triplicate are shown ± standard error of the mean. Significance was determined using a two-tailed, unpaired t test. *, p < 0.05 (No FAs vs a15:0/a17:0): †, p < 0.05 (No FAs vs 18:1Δ9). **A)** Percent growth of *S*. *aureus* in the presence of PYRC. **B)** Percent growth of *S*. *aureus* in the presence of EQI.

In bacteria, fatty acids are generated through the fatty acid synthase (FAS) pathways (S3 Fig) [12]. *S*. *aureus* utilizes the type II FAS system [20–22]. Saturated branched-chain fatty acids (SBCFAs) constitute a major component of the *S*. *aureus* membrane. When challenged with compounds that inhibit fatty acid synthesis (e.g. andrimid, an inhibitor of ACC), *S*. *aureus* overcomes inhibition by incorporating exogenous SBCFAs [20, 22]. This is attributed to availability of nonesterified acyl carrier proteins, which are otherwise depleted with FASII inhibitors in *S*. *aureus* [20–22]. SBCFAs can become incorporated into the membrane, thus maintaining membrane integrity [20–22]. In a study in which *S*. *aureus* was treated with fatty acid synthesis inhibitors, 50% of endogenous fatty acids were replaced following growth in media supplemented with oleic acid [22]. We found that SBCFAs (a15:0 and a17:0) and oleic acid (OA; 18:1Δ9) significantly rescued bacterial growth against PYRC and EQI treatments, consistent with the idea that PYRC and EQI target fatty acid biosynthesis (Fig 5). When EQI or PYRC were tested for activity against an acetyl-CoA carboxylase (ACC) deficient *S*. *aureus* mutant (Δ*aacD*; generously provided by Dr. Rock); no effect was observed. This was because the mutant also requires addition of a15:0 and a17:0 or OA to grow, and these same fatty acids rescue *S*. *aureus* from EQI inhibition. Thus, neither EQI nor PYRC exhibit growth inhibition in a condition in which ACC is absent.

### PYRC and EQI inhibit *de novo* fatty acid synthesis in *S*. *aureus*

**Fig 6:**
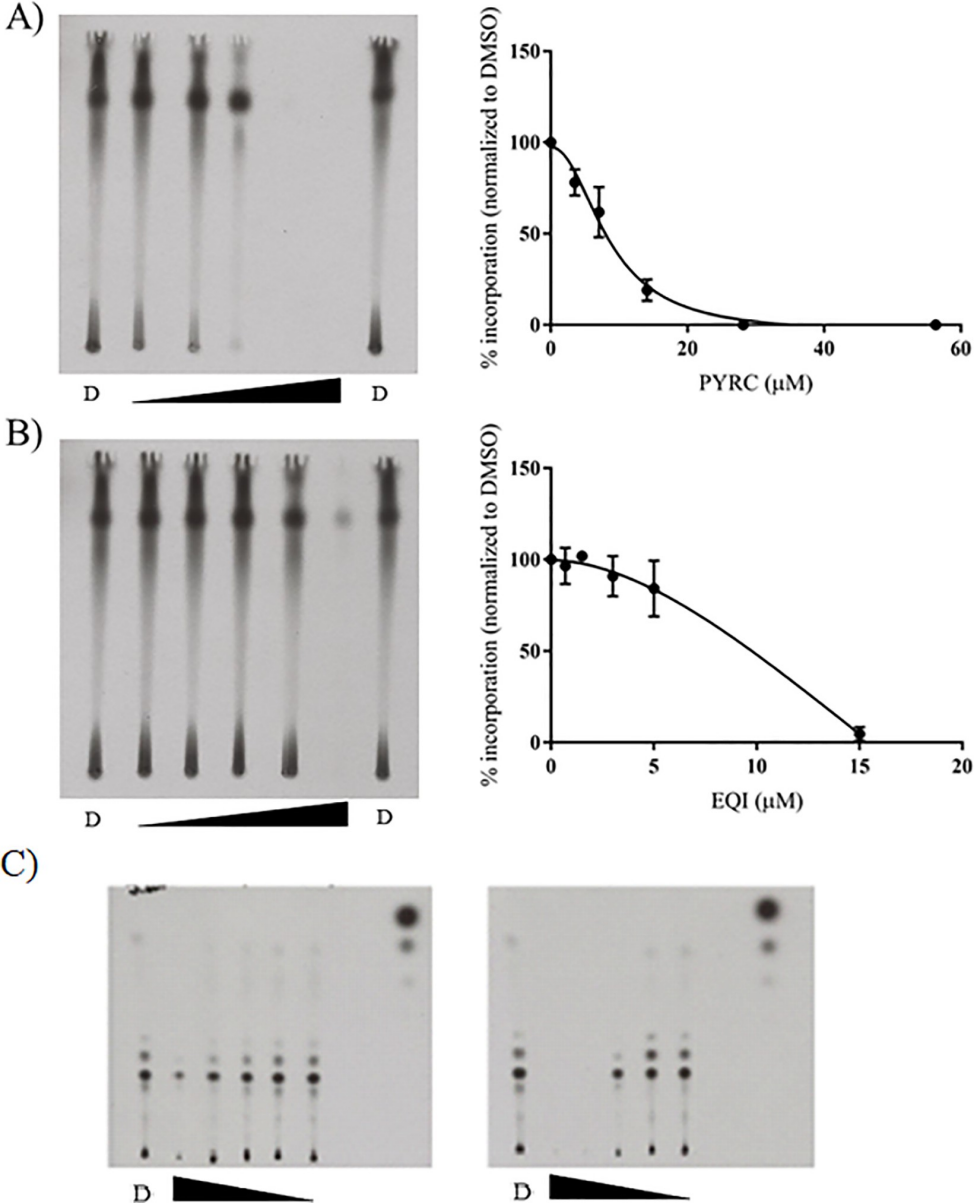
PYRC and EQI impair [1-^14^C] acetate incorporation in *S*. *aureus*. *S*. *aureus* was incubated with [1-^14^C] acetate (4μCi/mL) for 2 hours in the presence of DMSO, PYR or EQI. **A)** left panel, representative X-ray film of lipid extract from *S*. *aureus* following PYRC treatment eluted on a reverse-phase TLC plate. DMSO control indicated as “D”. The triangle indicates an increase in drug concentration from left to right. Right panel, percent incorporation of [1-^14^C] acetate following PYRC treatment normalized to DMSO. Mean data of two independent experiments are shown ± standard error of the mean. **B)** Left panel, representative X-ray film of lipid extract from *S*. *aureus* following EQI treatment eluted on a reverse-phase TLC plate. DMSO control indicated as “D”. The triangle indicates an increase in drug concentration from left to right. Right panel, percent incorporation of [1-^14^C] acetate following EQI treatment normalized to DMSO. Mean data of two independent experiments are shown ± standard error of the mean. **C**) Representative X-ray film of lipid extract from *S*. *aureus* following PYRC and EQI treatment eluted on a normal-phase TLC plate. DMSO control indicated as “D”. The triangle indicates a decrease in drug concentration from left to right. Tested concentrations of EQI: 0.7, 1.5, 3, 5, and 15 μM; PYRC 3.5, 7, 14, 28, and 56 μM. Left panel, percent incorporation of [1-^14^C] acetate following EQI treatment normalized to DMSO. Right panel, percent incorporation of [1-^14^C] acetate following PYRC treatment normalized to DMSO.

**Fig 7:**
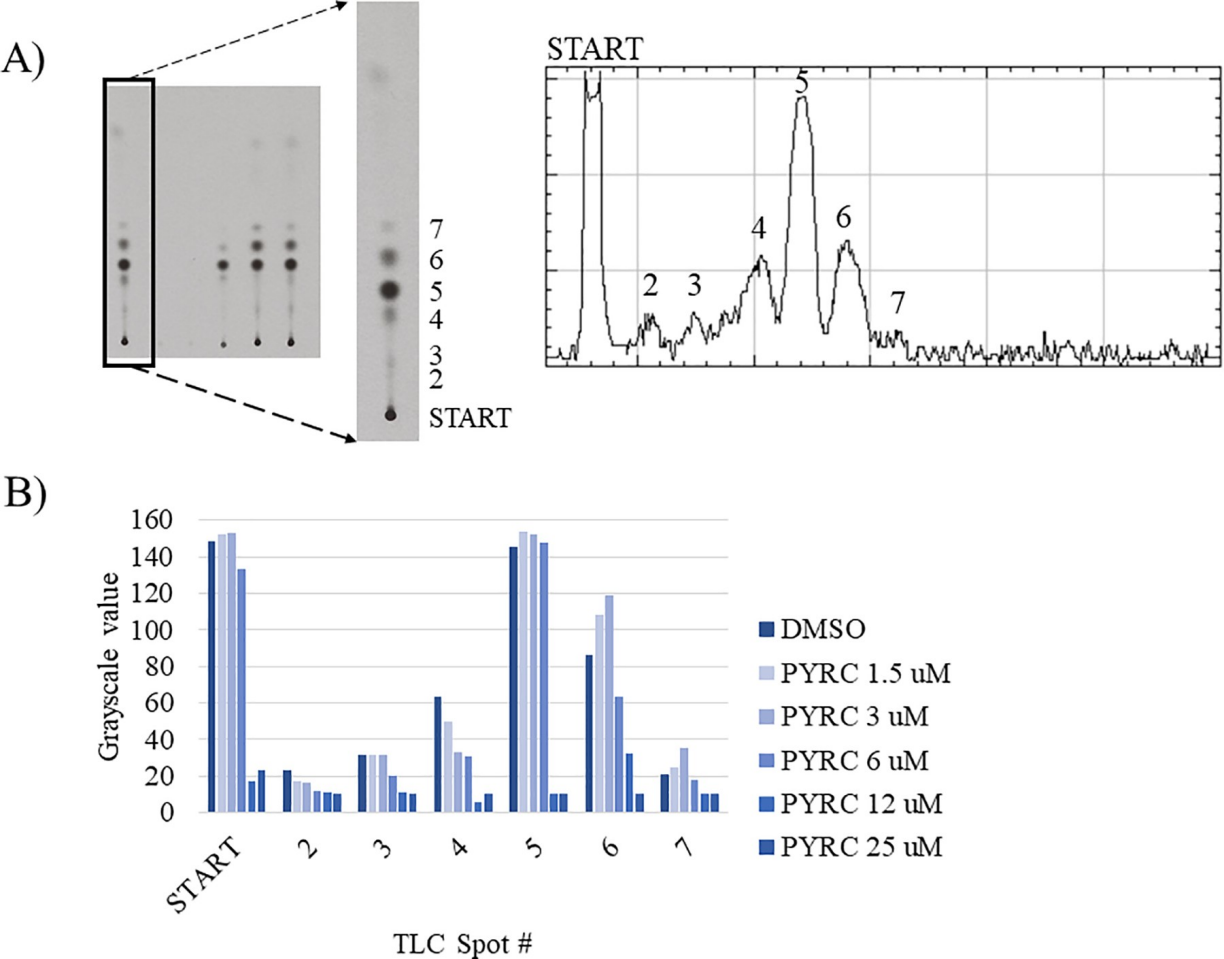
Densitometry scan of X-ray film from normal-phase TLC of incorporation of [1-^14^C] acetate into lipid fraction from *S*. *aureus* following PYRC treatment. Performed as in Fig 6C, right), above, and normalized to DMSO control. **A)** Densitometry scan of DMSO control lane on X-ray film, increasing R_f_, left to right. Spots quantified across PYRC concentrations included Start (origin) and discernable densitometry peaks (spots) 2–7. **B)** Relative grayscale of film exposure densities at the tested PYRC concentrations, 0, 1.5, 3, 6, 12 and 25 μM. Incorporation of radiolabeled [1-^14^C] acetate was decreased uniformly across the radiolabeled components of the extract in a dose-related fashion.

*De novo* fatty acid synthesis is initiated by ACC [20]. ACC is a multi-subunit enzyme in *S*. *aureus* that converts acetyl-CoA to malonyl-CoA via the addition of an activated carboxyl group. We used radioisotope tracing to test whether PYRC and EQI would inhibit incorporation of acetate into fatty acids of treated *S*. *aureus*. Radiolabeled acetate is a well-established tool to trace *de novo* fatty acid synthesis [22–24]. Upon entering the cell, acetate is converted into acetyl-CoA by acetyl-CoA synthase and utilized by subsequent pathways, including fatty acid biosynthesis. Dose-dependent inhibition of [1-^14^C]-acetate incorporation into chloroform:methanol (1:2) extractable-lipids [25] from *S*. *aureus* was observed with both PYRC and EQI, consistent with inhibition of acetyl-CoA carboxylase (Fig 6). When the chloroform:methanol extractable-lipids were analyzed by LCMS, a complex mixture of modified fatty acids, lipids and lipid soluble metabolites was identified. Because of the complex nature of lipid metabolism, it was not possible to quantify changes in individual components. However, when the TLC plate films were simply analyzed by densitometry, it was apparent that the all radiolabeled components of the mixture (represented by TLC spots), were broadly and proportionately diminished by EQI or PYRC in a dose dependent fashion, consistent with early inhibition of *de novo* fatty acid synthesis (Fig 7). Both normal phase and reverse phase TLC was used in order to take an unbiased approach in quantification of polar and nonpolar lipid fractions (Fig 6).

### PYRC and EQI inhibit biotin carboxylase catalytic domain of ACC

Based upon the hypothesis that ACC was inhibited, we tested the actions of EQI and PYRC against recombinant *E*. *coli* ACC *in vitro*. ACC catalyzes the first committed and regulated step in fatty acid biosynthesis [30]. The reaction catalyzed by ACC involves two half-reactions and is shown in Scheme 1. Bacterial ACC is composed of three proteins: biotin carboxylase (BC), carboxyltransferase (CT), and biotin carboxyl carrier protein (BCCP). In the first half-reaction, BC catalyzes an ATP-dependent carboxylation of biotin, which is covalently attached to BCCP. In the second half-reaction, CT transfers the carboxyl group from biotin to acetyl-CoA to produce malonyl-CoA. Biotin carboxylase and carboxyltransferase retain their activity when isolated from the other components and can utilize free biotin as a substrate [30].

$$\begin{array}{l} \mathrm{Half}‐\mathrm{reaction}\;1:\;\mathrm{BCCP}‐\mathrm{biotin}+\mathrm{MgATP}+{\mathrm{HCO}}_{3}\overset{BC}{\underset{Mg^{2+}}{⇌}}\mathrm{BCCP}‐\mathrm{biotin}‐CO_{2}{}^{‐}+\mathrm{MgADP}+\mathrm{Pi}\\ Half‐reaction\;2:\;BCCP‐biotin‐CO_{2}{}^{‐}+Acetyl‐CoA\overset{CT}{⇌}BCCP‐biotin+Malonyl‐CoA\\ \;\mathrm{Scheme}\;1.\;\mathrm{Reaction}\;\mathrm{catalyzed}\;\mathrm{by}\;\mathrm{acetyl}‐\mathrm{CoA}\;\mathrm{carboxylase}. \end{array}$$

**Fig 8:**
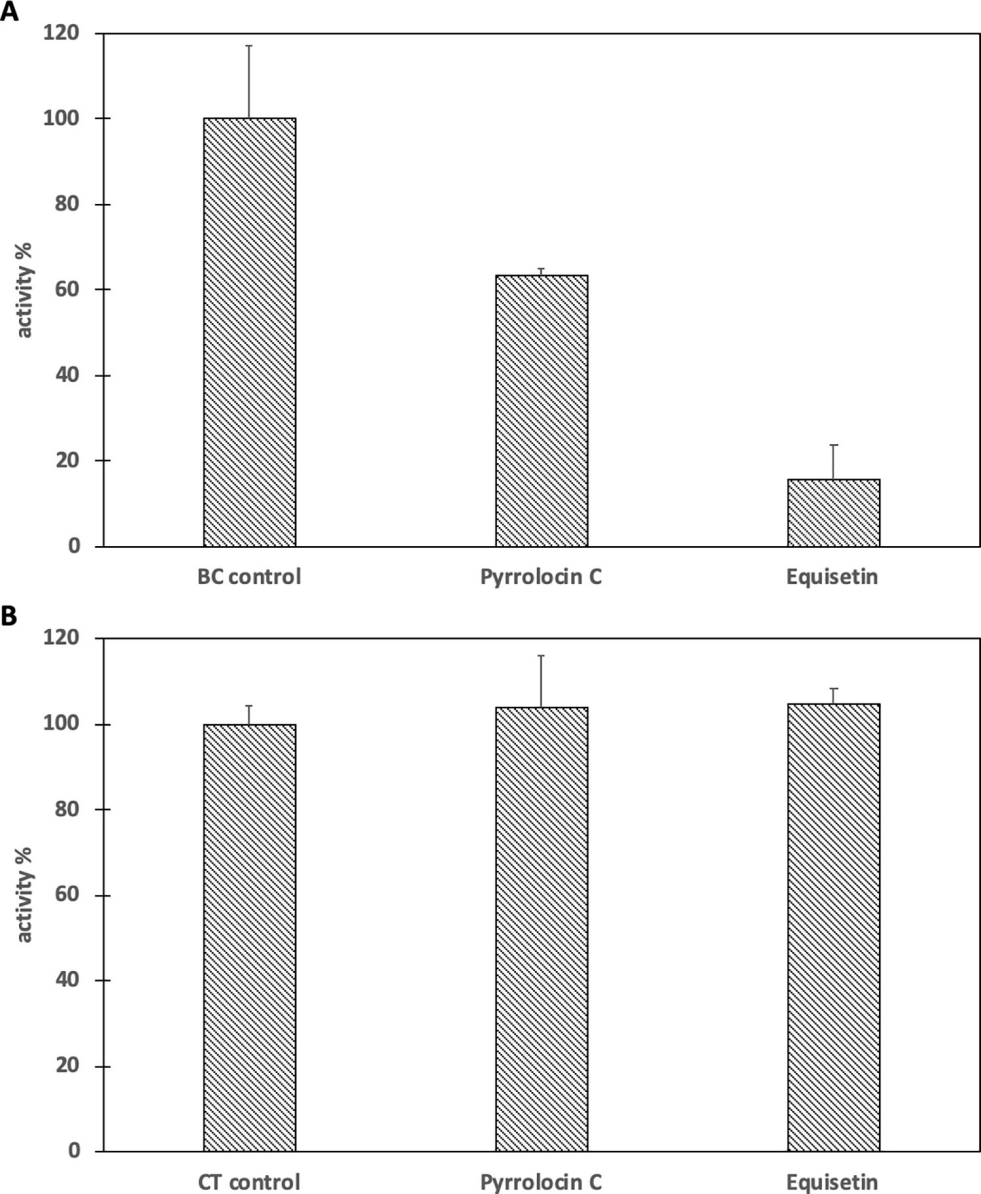
Inhibition of the biotin carboxylase (BC) and carboxyltransferase (CT) components of acetyl-CoA carboxylase by Pyrrolocin C and Equisetin. The concentration of each inhibitor was 0.1 mM. **A.** Percent activity of biotin carboxylase in the presence of Pyrrolocin C or Equisetin. The initial velocity of biotin carboxylase in the absence of inhibitor was measured and set to 100%. The substrates biotin (40 mM) and ATP (0.2 mM) were held constant at subsaturating levels. **B.** Percent activity of carboxyltransferase in the presence of Pyrrolocin C or Equisetin. The initial velocity of carboxyltransferase in the absence of inhibitor was measured and set to 100%. The substrates biocytin (6 mM) and malonyl-CoA (0.04 mM) were held constant at subsaturating levels. All assays were performed in triplicate ± S.D.

In order to determine which subunit of ACC is inhibited by PYRC and EQI, both subunits (BC and CT) were purified and assayed separately. PYRC and EQI inhibited biotin carboxylase by 36.6% and 84.5% respectively (Fig 8A). Control reactions showed neither PYRC nor EQI inhibited the coupling enzymes. Similarly, inhibition of CT was not observed by either PYRC or EQI (100μM each) (Fig 8B).

## Discussion

EQI and related tetramic acid natural products have long been recognized for their wide activity against eukaryotic and Gram-positive bacterial cells. Multiple lines of evidence indicate that EQI and related compounds exert their effects on eukaryotic cells by affecting mitochondrial metabolism [5, 6, 24, 26, 27]. The compounds also bind to metals efficiently and may affect metal homeostasis or metalloproteins. Indeed, HIV integrase is targeted by EQI based upon the metal-binding property [28]. For these reasons, tetramic acids have been considered to be general toxins that may not be useful as drugs. In this work, we show instead that EQI and PYRC act against *S*. *aureus* specifically by inhibiting fatty acid biosynthesis. Moreover, our data strongly suggest that malonyl-CoA synthesis by the ACC enzyme is the direct target of EQI and PYRC We sought to investigate PYRC because we noted promising activity against *M*. *tuberculosis* that differed in mechanism from other TB drugs (Fig 3). Based upon the previous data on EQI, we initially explored energy metabolism and metal homeostasis. Indeed, transcriptome analysis brought up a number of transcripts involved in energy utilization and metal cation binding (S1 Table). However, none of the energy protein transcripts could readily be related to decreased ATP levels (which we observed to trend toward a decrease in the metabolome study; Table 2) nor to signature transcripts connected to the observed inhibitory activity [10, 12]. In examining metal homeostasis by ICP-OES, we observed relatively large changes upon EQI treatment, but these were not tied to cell growth inhibition and could not be restored by addition of exogenous metals (Fig 4). Thus, we concluded that these effects were not likely central to PYRC or EQI mechanism of action.

We turned to metabolomics to further examine the effects of EQI on bacteria focusing on *S*. *aureus*, for which there is rich literature regarding its metabolism. When EQI was added at a sub-lethal dose to *S*. *aureus*, only a small number of metabolites showed a significant change. Among these, several were involved in the tricarboxylic acid cycle and the metabolic state of the cell, including a greatly increased amount of lactate. The increase in lactate could be explained by previous work on metal homeostasis in *S*. *aureus*, in which it was demonstrated that iron limitation led to an increase in fermentative metabolism [29].

Beyond central metabolism, the most profoundly decreased metabolite upon EQI treatment was malonyl-CoA, while acetyl-CoA was also modestly increased (Table 2). These data led us to suspect that fatty acid synthesis inhibition might play a role in the biological activity of EQI. Moreover, an increased acetyl-CoA pool would feed into the TCA cycle, potentially explaining some of the metabolomics results. To determine whether fatty acid synthesis was indeed inhibited by EQI and PYRC, we examined [1-^14^C]-acetate incorporation into growing fatty acids. Consistent with selective inhibition, *de novo* fatty acid synthesis was decreased in a dose-dependent manner that was similar to the MIC of EQI and PYRC. Furthermore, the attenuation of [1-^14^C]-acetate incorporation was observed to impact the wide variety of lipid containing metabolites fractionated by TLC approximately evenly.

Fatty acid biosynthesis begins with the conversion of acetyl-CoA into malonyl CoA by the ACC enzyme complex [30–34]. Subsequently, in *S*. *aureus* and most bacteria (with the exception of mycobacteria and a few others) a type II fatty acid synthase (FAS) converts malonyl-CoA into fatty acids. Previously, Rock and co-workers demonstrated that fatty acid synthesis inhibition in *S*. *aureus* leads to an accumulation of malonyl-CoA, which is toxic to the cell [35]. However, inhibition of acetyl-CoA carboxylation by the ACC enzyme complex diminishes malonyl-CoA, consistent with our metabolomics result. Rock and colleagues restored growth of *S*. *aureus* in the presence of an ACC inhibitor by adding exogenous fatty acids, but the growth was not reestablished by fatty acids in the presence of FAS inhibitors [35]. We treated *S*. *aureus* with EQI or PYRC in the presence or absence of exogenous fatty acids (a15:0/a17:0 and 18:1Δ9), demonstrating a recovery very similar to what was observed by Rock and colleagues in the presence of ACC inhibitor (Fig 5). These results implicated ACC as the target of the pyrrolidinedione acid metabolites, EQI and PYRC. Using purified *E*. *coli* ACC components, we demonstrated that the biotin carboxylation step of ACC catalysis was indeed inhibited by EQI and PYRC. Ongoing work is now focused on description of the inhibition kinetics using *S*. *aureus* ACC and determination of the crystal structure of EQI- and PYRC bound to *S*. *aureus* ACC.

ACC has emerged as an important drug target in both humans and in bacteria [11, 36]. Several selective compounds exist that target only the eukaryotic ACC [36]. Among the drugs targeting bacterial ACC, several are natural products, including thailandamide B, andrimid, and moiramide B [36–39]. Mutational analysis of *Salmonella enterica* ACC resistant to thailandamide B revealed a series of residues in the beta protein adjacent to the interface between the alpha and beta subunits [37, 38]. A crystal structure of moiramide B bound to the *S*. *aureus* ACC demonstrated that inhibition occurs at the interface between the alpha and beta subunits [39], which is nearby but not identical to the binding site proposed for thailandamide B.

Moiramide B and andrimid are 2,5-pyrrolidinedione- based natural products. It was shown that a beta-ketoamide / pyrrolidinedione was essential for activity; the beta-ketoamide was present in the enol tautomer, which bound in the same place that anion chemistry would take place during the catalytic cycle [14–16]. Similarly, tetramic acids are 2,4-pyrrolidinediones, with beta-ketoamides. The tautomerization of tetramic acids is essential to their bioactivity and metal binding activities and has been studied in detail [40, 41]. For example, the pKa of tetramic acids is ~2.5–4 [16]. Thus, we hypothesize that in tetramic acids, the pyrrolidinedione warhead could bind in the same place as in the moiramide B series. Alternatively, it is known that there is a zinc cation in the *S*. *aureus* ACC [39, 42]; perhaps metal chelation is again important in this instance. Further research is required to determine the exact molecular interactions by which EQI and PYRC inhibit ACC.

It should be emphasized that the pyrrolidinedione moiety is not sufficient to impart inhibition of fatty acid biosynthesis. A series of moiramide derivatives was synthesized, in which required structural features were identified [11, 26]. In the case of EQI and PYRC, very similar tetramic acid moieties are present. The methyl group of EQI is not essential, since it is absent in PYRC, and the methylated pyrrolocin A is nearly equipotent as an antibiotic to PYRC [4]. EQI and PYRC also have very similar 10-membered cyclic decalin subunits, as does TA-289 [6], which are decorated slightly differently with functional groups. Pyrrolocin B is much less active as an antibiotic, and its configuration at the decalin ring is *cis*, rather than *trans* as found in EQI and PYRC. Thus, the substituents attached to the tetramic acid moiety are highly important for specificity of action.

EQI and PYRC display approximately 1 log bacterial selectivity versus human cells in culture, which suggests that a safe therapeutic window may be achievable with this class of antibiotic. It is currently unclear what the molecular mechanism of action might be for EQI in eukaryotic cells. It is possible that the inhibition of fatty acid synthesis leads to mitochondrial dysfunction, as found in some known inhibitors [43]. Whether the target in eukaryotic cells is similar or much different, selective ACC inhibition suggests that these compounds may be modified to produce useful antibiotic agents. Moreover, recent data indicate that EQI can be formulated to act as a specific antibiotic agent in animals [44], and drug delivery of EQI as a topical application yielded reduced infection and increased wound healing [45]. Crucially, EQI and PYRC exhibit consistent activity against challenging, drug-resistant strains [46], including those resistant to methicillin, clindamycin, doxycycline and vancomycin. The novel mechanism of action may thus lead to utility in the ongoing battle against drug resistant infections.

## Materials and methods

### Reagents

*Staphylococcus aureus* (*S*. *aureus* subsp. *aureus* ATCC® 12600^TM^) and methicilin-resistant *S*. *aureus* (*S*. *aureus* subsp. *aureus* Rosenbach ATCC®43300^TM^; SCCmecA^+^) were obtained from ATCC (Manasus, VA). Clindamycin/Doxycycline-resistant *S*. *aureus* (*S*. *aureus* subsp. *aureus* CDC-568; tet(K), aplh(3’)-III) and vancomycin-resistant *Enterococcus faecalis* (*E*. *faecalis* ATCC®51299^TM^; vanB^+^) were kindly donated by Dr. Ryan Looper of the University of Utah. *Escherichia coli* (ATCC®23724^TM^) were kindly donated by Dr. Matthew Mulvey of the University of Utah. Nutrient agar and Mueller Hinton broth were obtained from Difco (cat. # 211665) and Sigma-Aldrich (cat. # 70192), respectively. Luria Bertani (LB) broth was obtained through Fisher Scientific (cat. # 22700–025). Oxacilin sodium salt was obtained from Sigma-Aldrich (cat. # 28221). Polymyxin B and polymyxin B nonapeptide were obtained from Sigma-Aldrich (cat. # P4932-5MU and P2076-5MG, respectively). [1-^14^C] Acetate was obtained from American Radiolabeled Chemicals, Inc. (specific activity, 55mCi/mmol) and Carestream®Kodak®Biomax® MR Film from Sigma-Aldrich (cat. # Z350370-50EA). Acetone, chloroform, hexane, methanol, dimethyl sulfoxide and isopropyl alcohol were obtained through Fisher Scientific (Waltham, MA). Oleic acid was purchased from Acros Organics (AC270290050). Anteisopentadecylic acid (a15:0) and anteisomargaric acid (a17:0) were obtained through Ultra Scientific (FLBA-026 and FLBA-027, respectively; North Kingston, RI). Bovine serum albumin (lyophilized powder, essentially fatty acid free) was obtained through Sigma-Aldrich (cat. # 60757). Thin liquid chromatography silica gel 60 F_254_ (cat. # 5554–7; EMD Chemicals). Thiazolyl blue tetrazolium bromide (MTT) was purchased through Sigma-Aldrich (cat. # M2128-1G).

### Antimicrobial microdilution assay

Glycerol stocks of *S*. *aureus*, doxycycline/clindamycin-resistant *S*. *aureus*, and vancomycin-resistant *E*. *faecalis* were thawed and streaked on Nutrient agar (NA) plates. Methicillin-resistant *S*. *aureus*, on the other hand, was streaked on Nutrient agar supplemented with 8μg/mL oxacillin. *E*. *coli* was streaked on Luria-Bertani agar plates. Plates were incubated at 37°C for 8 – 12h. A single colony from the NA plates was transferred into 7mL Mueller Hinton broth (MHB) (with additional 2% NaCl for MRSA)(LB broth for *E*. *coli*) and incubated for 6-8h at 30°C, 150 rpm. The turbidity of the broth culture was then adjusted to match the 0.5 McFarland standard (1 x 10^8^ cells/mL). The adjusted broth culture was then further diluted 200-fold and used as the final inoculum for the assay. 200 μL of the test organism was dispensed to each well of a 96 well plate. PYRC, EQI, positive control and 1% DMSO as solvent control were tested using a two-fold dilution scheme starting at 32μg/mL with 10 dilutions each. For *E*. *coli*, PYRC (2–128 μg/mL), EQI (2–128 μg/mL), polymyxin B (0.016–1 μg/mL) and polymyxin B nonapeptide (0.125–64 μg/mL) were tested singly, and in combination using a two-fold dilution scheme. Following an 18 – 20h incubation period, ten microliters of MTT (5mg/mL) were added to wells and incubated for 2h. One hundred microliters of solubilizing reagent (50% DMF/20% SDS) was added to wells and incubated for 1 hour. The A_570_ was measured using a Biotek–Synergy 2 Microplate Reader (Biotek;Winooski, VT).

### Isolation of PYRC

The heterologous expresser, *Fusarium heterosporum* ΔpyrG:Peqx:prlS+prlC, was used to generate PYRC [5]. *F*. *heterosporum* ΔpyrG:Peqx:prlS+prlC spores were added to corn grit agar (CGA) and grown for 21 days at 25°C. CGA flasks were extracted twice with acetone and dried down *en vacuo* onto C_18_-reversed phase silica gel (cat. # 60757; Sigma-Aldrich). PYRC was semi-purified by flash chromatography through 10% stepwise increases in acetonitrile in water. Individual peaks were isolated by reverse-phase HPLC (Phenomenex Luna 5u C18(2); cat. # 00G-4252-NO). PYRC was confirmed by LC-MS and H-NMR.

### Transcriptomic analysis of PYRC and prototypical anti-TB drugs

H37Ra was grown to log phase, measured by OD_600_. An aliquot of the bacterial culture was added to 7H9 media to achieve a final volume of 50 mL. The following drugs were added to the cultures, in triplicate, to result in the final concentrations (~10X minimum inhibitory concentration or IC90): rifampin (RIF; 12.2 μM), isoniazid (INH; 14.6 μM), pyrazinamide (PZA; 812 μM), ciprofloxacin (CIP; 30.2 μM), gentamicin (GEN; 20.9 μM), *p*-aminosalicylate (PAS; 71.8 μM), PYRC (PYRC; 225 μM, determined in Ref. 6), and DMSO (vehicle). Cultures were incubated at 37°C for 6 hours. The bacteria were pelleted and washed with phosphate buffered saline (PBS). RNA from the bacterial pellet was extracted using the Zymo Research (ZR) Fungal/Bacterial RNA MiniPrep™ Kit (cat. # R2014; Irvine, CA), as per instructions with an additional on-column Invitrogen™ Ambion™ TURBO™ DNase I digestion (cat. # AM2238, 2U/uL; Waltham, MA). RNA was submitted to the University of Utah High-Throughput Genetics Sequencing Core Facility for total RNA analysis by Illumina HiSeq. Data was assembled by the University of Utah Bioinformatics Core Facility. Differential expression analysis and principal component analysis of drug treatments versus vehicle (DMSO) were performed using Partek (Partek, Inc.; St. Louis, MO). Differential expression analysis of drug compared to vehicle (DMSO) generated a gene list that were significantly upregulated or downregulated (fold-change: -2 ≥ x ≥ 2; p < 0.05). These gene lists were analyzed using functional clustering analysis in the National Institute of Allergy and Infectious Diseases DAVID website [13].

### Total metal analysis of EQI-treated *S*. *aureus* by ICP-OES

An aliquot of an overnight *S*. *aureus* culture was added to LB broth, followed immediately with DMSO or EQI (4.36 μM). Cultures were incubated at 37°C for 2 hours. Bacteria were pelleted and washed in 1X phosphate buffered saline (PBS). Bacterial pellets were digested in 70% nitric acid (trace metal grade) at 100°C for 15 mins. Digestates were diluted with MilliQ water and submitted for ICP-OES analysis at the University of Utah Iron & Heme Core Facility. Total metal concentrations were adjusted based on final OD_600_.

### Exogenous metal rescue of EQI-treated *S*. *aureus*

*S*. *aureus* cultures were incubated in DMSO or EQI (4.36 μM) in the presence or absence of exogenous metals at 37°C for 2 hours. Metal solutions (MgCl, MnCl, & Fe(EDTA)) were added to reach the following concentrations: 1 μM, 10 μM, and 100 μM. Percent survival was determined by the MTT assay.

### Metabolomic analysis of EQI in *S*. *aureus*

An aliquot of an overnight *S*. *aureus* culture was added to LB broth, followed immediately with DMSO or EQI (4.36 μM). Two sets of cultures were prepared in quintuplicate for DMSO and EQI (one set for the acidic preparation and one set for the basic preparation). Cultures were incubated at 37°C for 2 hours. The final OD_600_ was measured for each culture following the 2 hours. The bacteria were pelleted at 12000 x g, 4°C for 10 min and washed with PBS. Bacterial pellets were reconstituted in cold 90% MeOH/1% formic acid (acidic preparation) or 90% MeOH/1% ammonium hydroxide (basic preparation). Preparations were bead milled on high for 2 min. Water was added to bring MeOH concentration to 50%. The preparations were pelleted at 12000 x g, 4°C for 10 min. The supernatant was transferred and dried down *en vacuo*. Samples were submitted to the University of Utah Metabolomics Core Facility. Data was normalized to final OD_600_.

All LC-MS was performed using an Agilent 6550 QTOF fitted with an Agilent 1290 HPLC. A 100 x 2.1 mm, 3.5 μm, 100A SeQuantz zic-HILIC column (Merck Millipore, Darmstadt, Germany) was used for separation. Ten millimolar ammonium carbonate (pH 9) was used as the strong solvent with acetonitrile the weak solvent. The initial LC condition was held at 90% acetonitrile for one min followed by a 20-min ramp to 40% acetonitrile and a 2-min hold. The column was re-equilibrated over 10 min at 90% acetonitrile. Flow rate was 0.2mL/min. Data was recorded and translated to file format using MassHunter software (Agilent, Santa Clara CA).

### Fatty acid rescue

*S*. *aureus* was streaked onto nutrient agar plates containing ethanol (EtOH), oleic acid (500 μM, constituted in EtOH), and a15:0/a17:0 (500 μM; 2:1 ratio of a15:0 to a17:0, constituted in EtOH). Colonies from nutrient agar plates were added to LB broth containing BSA (10 mg/mL), BSA with oleic acid (500 μM), or BSA with a15:0/a17:0 (500 μM). Cultures were grown overnight at 37°C. Aliquots of overnight *S*. *aureus* cultures were added to LB broth containing either BSA, BSA with oleic acid, or BSA with a15:0/a17:0. Vehicle (DMSO) or drugs were added at the following concentrations: EQI (0.7, 1.5, 3.0, 5.0, and 15.0 μM) and PYRC (3.5, 7.0, 14.0, 28.1, and 56.3 μM) to 96-well plates to which *S*. *aureus* cultures were subsequently added. OD_600_ was measured after 14h using a Biotek–Synergy 2 Microplate Reader. Final OD_600_ was used to calculate % growth.

### [1-^14^C] Acetate incorporation

An aliquot of an overnight culture of *S*. *aureus* was added to LB broth to yield an approximate initial OD_600_ of 0.4. The culture was split for drug treatments with and without [1-^14^C] acetate. [1-^14^C] Acetate was added (4μCi/mL) and incubated at 37°C for 2 hours. Duplicate cultures without [1-^14^C] acetate was performed in parallel to normalize based on final OD_600_. Lipid fractions were extracted using a modified Bligh-Dyer method [25]. The bacteria were pelleted for 10 min at 12000 x g, 4°C, washed with PBS, and pelleted again. The pellet was extracted by reconstituting in a chloroform to methanol mixture (1:2). Additional chloroform and water was added for a final ratio of chloroform:methanol:water of 2:2:1. The chloroform layer was transferred and evaporated. Chloroform volume was adjusted based on final OD_600_ prior to loading onto normal or reverse-phase TLC plates. Hexane:acetone (25:4) developing solvent was used to develop normal-phase TLC plates and chloroform:methanol (3:2) was used to develop reverse-phase TLC plates. Carestream®Kodak®Biomax® MR film was exposed to TLC plates for 7 days and then developed using a Mini-Medical 90 Processor (cat. # 9992305300-U; AFP Imaging, Elmsford, NY). Grayscale values of film images were analyzed in Fiji [47]).

### LC-MS analysis of lipid fractions

A modified Bligh-Dyer method was used to extract the lipid fraction from bacteria cultures [25]. In brief, the bacteria were pelleted for 10 min at 12,000 × g, 4°C, washed with PBS, and pelleted again. The pellet was extracted by re-constituting in a chloroform to methanol mixture (1:2) and then shaken for 16 hours at room temperature. Additional chloroform and water was added for a final ratio of chloroform:methanol: water of 2:2:1. The mixture was centrifuged to separate the methanol/water layer and chloroform layer. The chloroform layer was removed, mixed with 10 grams of silica gel, and dried *en vacuo*. The lipid fraction was fractionated further with the use of a silica gel flash column. Three column volumes of hexane: acetone (25:4) was passed through the column and individual fractions were collected, followed by 2 column volumes of hexane:acetone (50:50) and 1 column volume of hexane:acetone (10:90). Fractions were spotted on a normal-phase TLC plate and visualized by charring (10% CuSO4/8% phosphoric acid) to determine retention factor of contents. These fractions were dried down *en vacuo* and reconstituted in 100% isopropyl alcohol. The samples were submitted to University of Utah Mass Spectrometry Core for lipidomic analysis.

### Protein purification and enzymatic assays

*E*. *coli* biotin carboxylase with a His-tag on the N-terminus was overexpressed and purified according to Blanchard et al. [48]. Carboxyltransferase with a His-tag on the N-terminus of the α-subunit was expressed and purified according to Blanchard & Waldrop [49].

The activity of biotin carboxylase was determined according to the method of Blanchard et al. [48]. The activity of carboxyltransferase was determined in the reverse non-physiological direction according to Blanchard & Waldrop [49].

### Statistical analysis

Unpaired t tests were performed on each analyte identified by metabolomic analysis (in quintuplicate). For the fatty acid residue experiments, unpaired t tests were performed comparing conditions without fatty acid to conditions with fatty acids across 3 independent experiments run in triplicate. Significance was determined if the p-value was less than 0.05.

## Supporting information

S1 Fig Minimum inhibitory concentration of **A)** pyrrolocin B, **F)** PYRC, and **K)** EQI against *S*. *aureus*. Minimum inhibitory concentration of **B)** pyrrolocin B, **G)** PYRC, and **L)** EQI against methicillin-resistant *S*. *aureus*. Minimum inhibitory concentration of **C)** pyrrolocin B, **H)** PYRC, and **M)** EQI against clindamycin & doxycycline-resistant *S*. *aureus*. Minimum inhibitory concentration of **D)** pyrrolocin B, **I)** PYRC, and **N)** EQI against vancomycin-resistant *E*. *faecalis*. IC_50_ against CEM-TART cells treated with **E)** pyrrolocin B, **J)** PYRC, and **O)** EQI. Bacterial strains listed in Table 1.
(TIF)

S2 Fig Potentiation of PYRC when combined with polymyxin B nonapeptide against E. coli (ATCC 23724). Heat map depicts percent inhibition (light blue;lower percent inhibition–dark blue; higher percent inhibition). Numbers inside the table are percent inhibition values.
(TIF)

S3 Fig Fatty acid biosynthesis in *Mycobacterium tuberculosis* and *Staphylococcus aureus*. (TIF)

S1 Table PYRC treatment induces pathways enriched in energy metabolism in Mtb. (DOCX)
