# Supplementary figures and images for: Pyrrolocin C and equisetin inhibit bacterial acetyl-CoA carboxylase
Source: PLoS One. 2020 May 29;15(5):e0233485. doi: 10.1371/journal.pone.0233485 (PMC7259786; doi:10.1371/journal.pone.0233485)

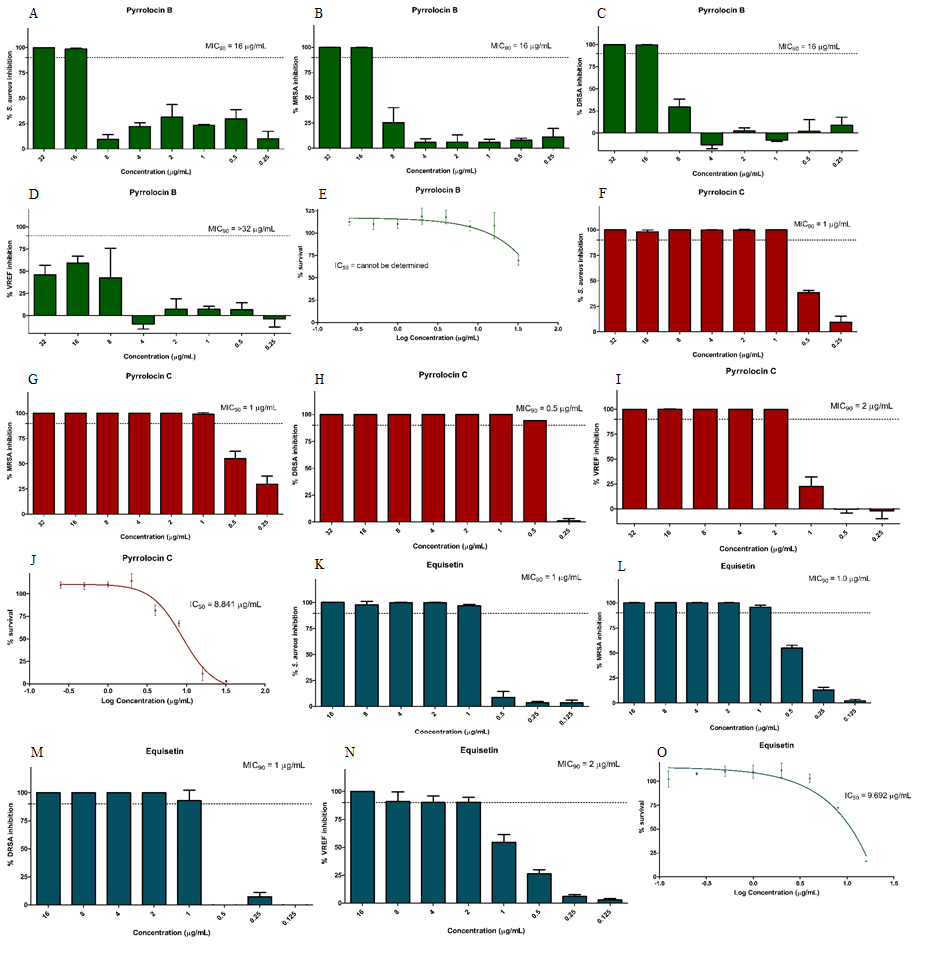

Supplement: S1 Fig — Minimum inhibitory concentration of A) pyrrolocin B, F) PYRC, and K) EQI against S. aureus. Minimum inhibitory concentration of B) pyrrolocin B, G) PYRC, and L) EQI against methicillin-resistant S. aureus. Minimum inhibitory concentration of C) pyrrolocin B, H) PYRC, and M) EQI against clindamycin & doxycycline-resistant S. aureus. Minimum inhibitory concentration of D) pyrrolocin B, I) PYRC, and N) EQI against vancomycin-resistant E. faecalis. IC50 against CEM-TART cells treated with E) pyrrolocin B, J) PYRC, and O) EQI. Bacterial strains listed in Table 1. (TIF) [file pone.0233485.s001.tif]

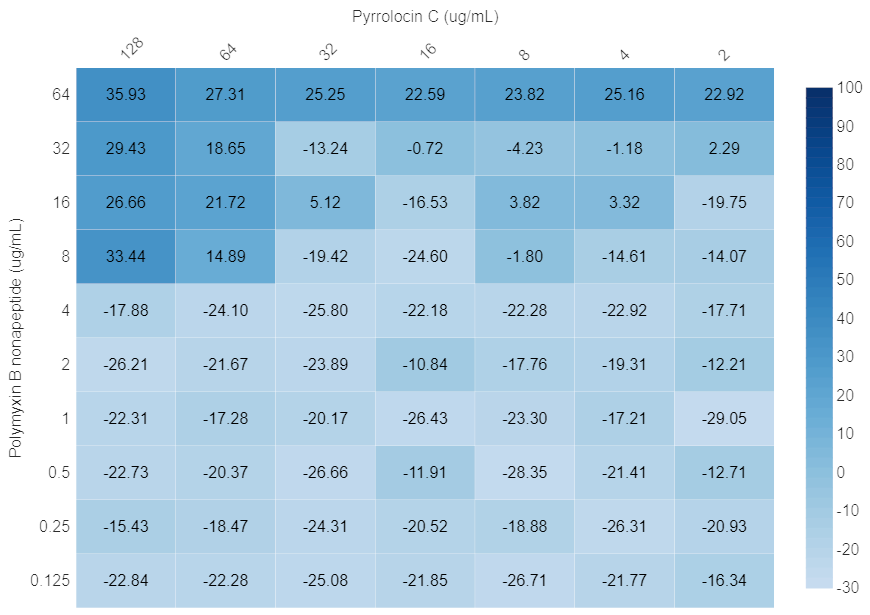

Supplement: S2 Fig — Heat map depicts percent inhibition (light blue;lower percent inhibition–dark blue; higher percent inhibition). Numbers inside the table are percent inhibition values. (TIF) [file pone.0233485.s002.tif]

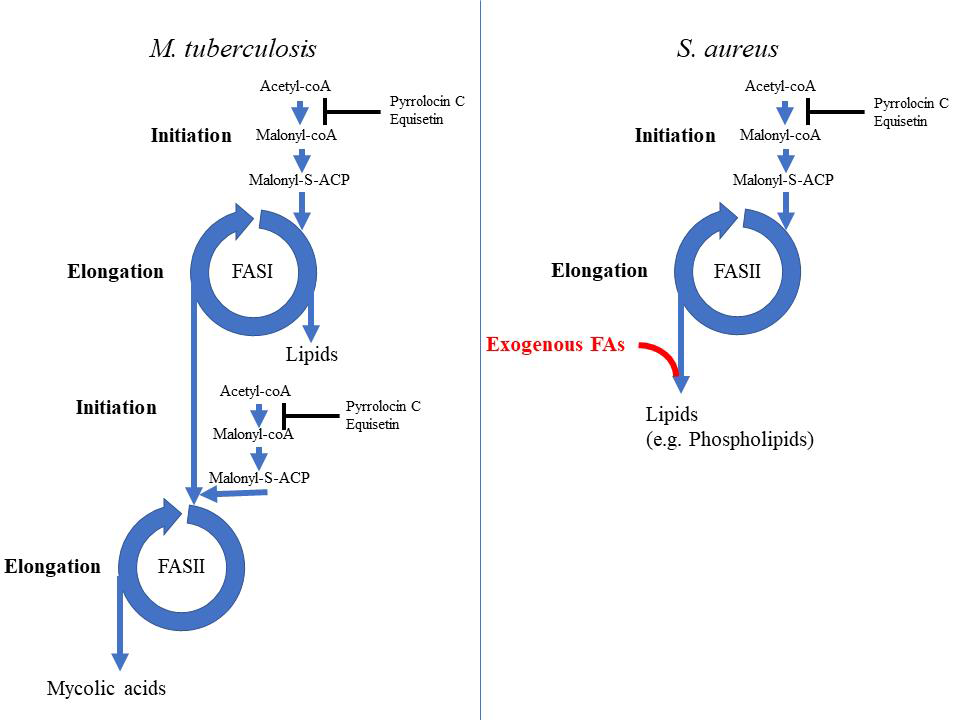

Supplement: S3 Fig — (TIF) [file pone.0233485.s003.tif]

**Table SI 1. PYRC treatment induces pathways enriched in energy metabolism in Mtb.
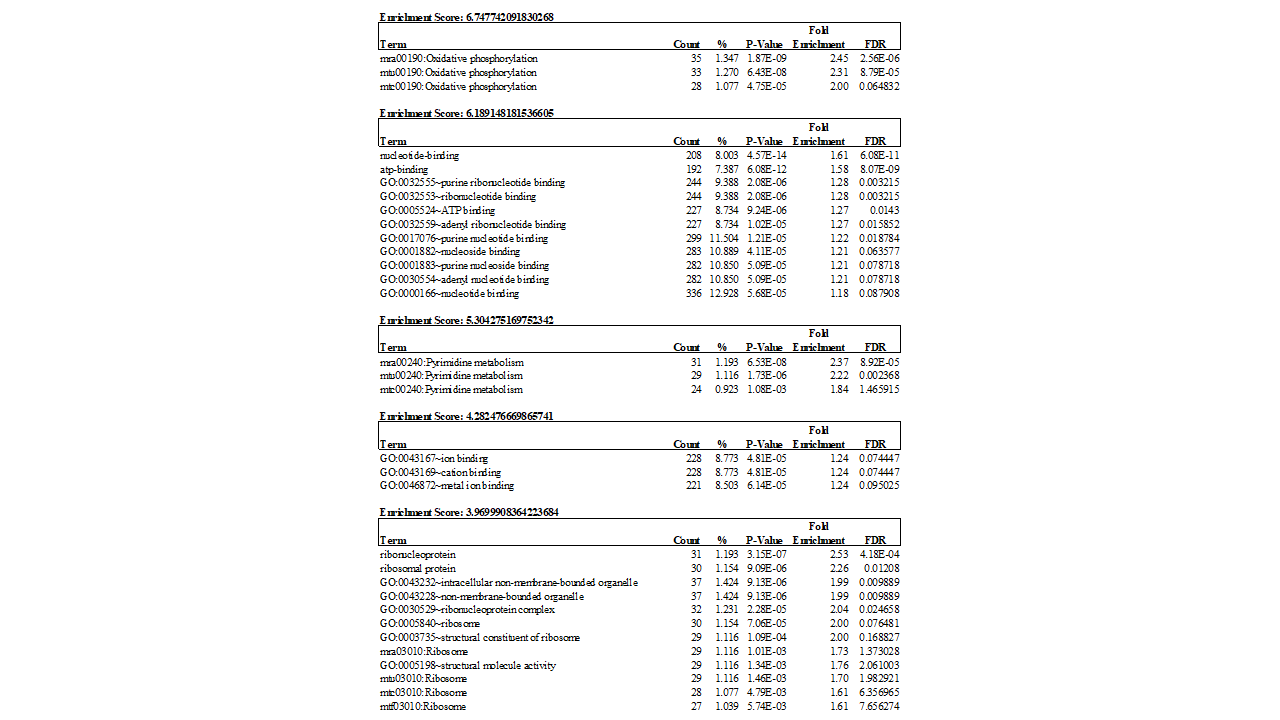
**

Supplement: S1 Table — (DOCX) [file pone.0233485.s004.docx]
